# Recombinant GII.Pe-GII.4 Norovirus, Thailand, 2017–2018

**DOI:** 10.3201/eid2508.190365

**Published:** 2019-08

**Authors:** Watchaporn Chuchaona, Jira Chansaenroj, Nasamon Wanlapakorn, Sompong Vongpunsawad, Yong Poovorawan

**Affiliations:** Chulalongkorn University, Bangkok, Thailand

**Keywords:** gastroenteritis, human norovirus, recombinant GII.Pe-GII.4, VP1, polymerase, viruses, Thailand, RT-PCR, enteric infections

## Abstract

During June 2017–December 2018, norovirus was responsible for 10.9% of acute gastroenteritis cases in Thailand. Genogroup I (GI) was found in 14% of samples, of which 12 were co-infected with genogroup II (GII). In 35.8% of samples, GII.Pe-GII.4 Sydney predominated. Diverse recombinant strains of GI and GII norovirus co-circulated year-round.

Norovirus is a major cause of nonbacterial acute gastroenteritis; sporadic cases and outbreaks occur among children and adults (*1*). Most strains infecting humans belong to genogroups I and II (GI and GII), of which GII.4 has been most predominant (*2*). Reemergence of norovirus infection is attributed to new variants resulting from frequent recombination between the end of open reading frame (ORF) 1, encoding the RNA-dependent RNA polymerase (RdRp), and ORF2, encoding the major capsid protein (VP1) of the norovirus genome. Lack of a robust cell culture system for human norovirus infection in humans and lack of long-lasting neutralizing antibodies in previously infected persons present challenges for vaccine development.

In recent years, a relative increase in global prevalence of norovirus has been attributed to GII.P16-GII.4, GII.17, and GII.P16-GII.2 (*3*–*5*). As in temperate regions, norovirus infection in Thailand occurs as sporadic cases and outbreaks year-round. After the recent increase of GII.P16-GII.2 in Thailand in late 2016 (*6*), we sought to identify the most frequently identified genotype(s).

During June 2017–December 2018, we tested 2,704 fecal/rectal swab samples from patients with acute gastroenteritis (watery diarrhea and sometimes vomiting) in hospitals in Bangkok (King Chulalongkorn Memorial Hospital and Bangpakok 9 International Hospital; n = 2,385 patients), Khon Kaen (n = 70), Phitsanulok (n = 199), and Saraburi (n = 50), Thailand. Mean patient age ± SD was 27.9 ± 25.1 years (range 1 month–103 years). These samples were collected for diagnostic tests routinely ordered by clinicians and served as convenient research samples; most (>95%) patients were inpatients. The institutional review board of Chulalongkorn University approved this study (IRB 634/59). 

We extracted viral RNA from 10% (wt/vol) fecal suspension with phosphate-buffered saline by using Ribospin vRD II (GeneAll, http://www.geneall.com) and subjected it to TaqMan Fast Virus 1-Step real-time reverse transcription PCR (RT-PCR) (Thermo Fisher, https://www.thermofisher.com) (*7*). We dual typed norovirus-positive samples by using RT-PCR to amplify the partial RdRp and VP1 genes (*6*) (GenBank accession nos. MK589361–402, MK590421–687, and MK590696–962). For genotyping, we used the Norovirus Genotyping Tool (http://www.rivm.nl/mpf/norovirus/typingtool). We phylogenetically analyzed nucleotide sequences by using the maximum-likelihood method with 1,000 bootstrap replicates in MEGA7 (http://www.megasoftware.net).

A total of 296 (10.9%) of 2,704 samples were positive for norovirus (patient mean age ± SD 14.8 ± 19.9 years, range 3 months–88 years). A minority of strains (42/296) were genotype GI (GI.3 15/42, GI.5 14/42, GI.1 6/42, GI.7 6/42, GI.4 1/42) (Appendix Figure 1). Most positive samples (266/296) were GII strains, of which 12 were co-infected with GI/GII. The 3 most common RdRp genotypes were GII.Pe (40.6%, 108/266), GII.P16 (28.6%, 76/266), and GII.P17 (16.2%, 43/266) (Figure, panel A). Among samples for which VP1 genotyping was successful, most were GII.4 Sydney (56.8%, 151/266), followed by GII.17 (16.2%, 43/266), GII.6 (5.3%, 14/266), GII.3 (5.3%, 14/266), GII.13 (4.9%, 13/266), GII.2 (4.1%, 11/266), and others (7.5%, 20/266) (Figure, panel B). The most prevalent strains were recombinant GII.Pe-GII.4 Sydney (39.9%, 106/266), GII.P16-GII.4 Sydney (17%, 45/266), and GII.P17-GII.17 (15%, 39/266). Although RdRp genotype GII.Pe is usually associated with VP1 genotype GII.4 Sydney, we identified 1 strain each of GII.Pe-GII.3 and GII.Pe-GII.13.

**Figure:**
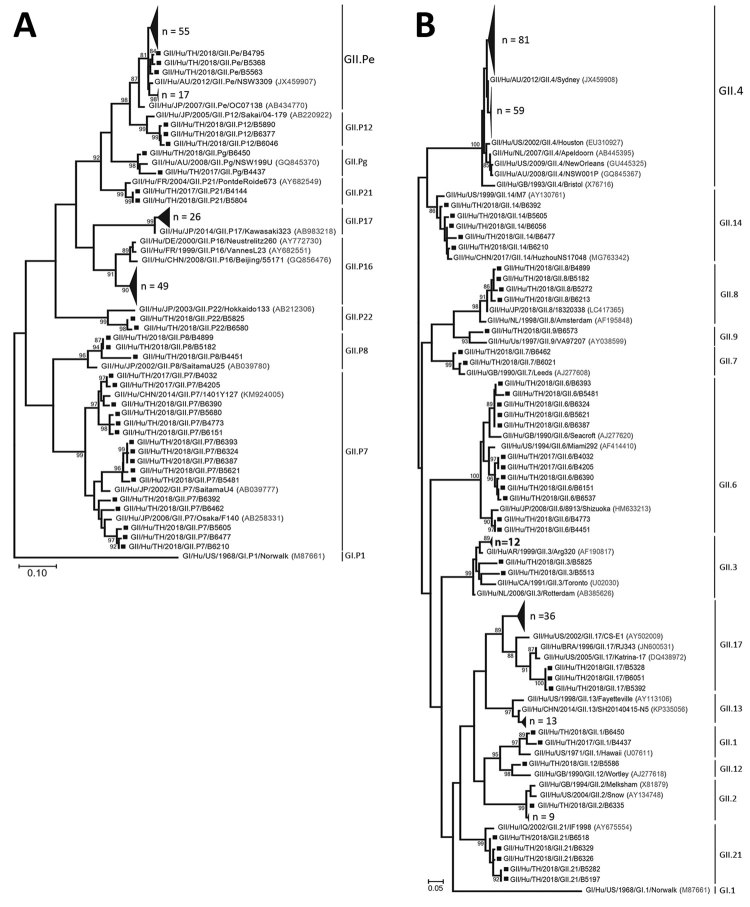
Phylogenetic trees of the norovirus GII partial-nucleotide sequences. A) Analysis of the RNA-dependent RNA polymerase (RdRp) region (380 bp). B) Analysis of the major capsid protein VP1 region (271 bp). Trees were generated by using the maximum-likelihood method with 1,000 bootstrap replicates implemented in MEGA7 (https://www.megasoftware.net). Bootstrap values >80 are indicated at the nodes. Strains of sufficient nucleotide sequence length were included in the trees (denoted individually with squares and in groups with large triangles). Reference strains are shown with accession numbers (in parentheses). Scale bars indicate nucleotide substitutions per site.

In this study, norovirus infections were frequently detected in children <5 years of age (60.1%, 178/296, mean age ± SD 2.2 ± 1.3 years). Using cycle threshold (C_t_) as a surrogate for viral load, we age stratified norovirus-positive patients (GII.Pe-GII.4 Sydney) into 3 age groups, <2, 2–5, and >5 years (Appendix Figure 2). Mean viral load was higher among children <5 years of age (C_t_ = 19.0) than among those >5 years of age (C_t_ = 25.1) (p<0.01).

In Thailand, the most frequently identified norovirus genotype throughout 2018 was GII.Pe-GII.4 Sydney. In the United States, GII.Pe-GII.4 Sydney first emerged in 2012 and was prevalent until mid-2015 (*3*,*8*); however, it represented minor variants elsewhere and has not been previously associated with increased norovirus activity in Thailand (*9*). Although GII.P16-GII.4 Sydney subsequently emerged in many industrialized countries, both GII.Pe-GII.4 Sydney and GII.P16-GII.4 Sydney circulated concurrently in Thailand after the upsurge of GII.17 in 2015–2016 and GII.P16-GII.2 in 2016–2017 in Thailand (*6*). Three years later, GII.17 continued to be detected sporadically, albeit at low levels. Therefore, norovirus circulation in Thailand at times differed from the global trend, which cannot be explained by factors such as geographic location and warm climate alone.

The significance of GI/GII co-infection in some samples is unclear but is probably not the result of laboratory contamination because as many as 7 combinations were identified (4 samples of GI.5/GII.17; 2 each of GI.3/GII.P16-GII.4 Sydney and GI.5/GII.P16-GII.21; and 1 each of GI.1/GII.P8-GII.6, GI.3/GII.17, GI.7/GII.17, and GI.7/GII.P7-GII.6). We did not perform dual typing on GI strains because they were less predominant than GII strains. However, future analysis of their recombination patterns will be useful for better characterizing these rare but potentially significant genotypes. This study was somewhat limited by lack of detailed clinical information accompanying the submitted samples and absence of surveillance from southern Thailand (≈14% of the country’s population). Molecular epidemiology and continued surveillance of norovirus strain diversity will increase awareness among clinicians and help epidemiologists determine global transmission patterns.

Appendix Phylogenetic tree of the norovirus GI partial nucleotide sequence of major capsid protein VP1, and cycle threshold values among patients in study of recombinant GII.Pe-GII.4 norovirus, Thailand, 2017–2018.

## References

[1] Ahmed SM, Hall AJ, Robinson AE, Verhoef L, Premkumar P, Parashar UD, et al. Global prevalence of norovirus in cases of gastroenteritis: a systematic review and meta-analysis. Lancet Infect Dis. 2014;14:725–30. 10.1016/S1473-3099(14)70767-4 24981041 PMC8006533

[2] Vinjé J. Advances in laboratory methods for detection and typing of norovirus. J Clin Microbiol. 2015;53:373–81. 10.1128/JCM.01535-14 24989606 PMC4298492

[3] Chan MCW, Hu Y, Chen H, Podkolzin AT, Zaytseva EV, Komano J, et al. Global spread of norovirus GII.17 Kawasaki 308, 2014–2016. Emerg Infect Dis. 2017;23:1359–1354. 10.3201/eid2308.161138 28726618 PMC5547775

[4] Niendorf S, Jacobsen S, Faber M, Eis-Hübinger AM, Hofmann J, Zimmermann O, et al. Steep rise in norovirus cases and emergence of a new recombinant strain GII.P16-GII.2, Germany, winter 2016. Euro Surveill. 2017;22:30447. 10.2807/1560-7917.ES.2017.22.4.30447 28181902 PMC5388089

[5] van Beek J, de Graaf M, Al-Hello H, Allen DJ, Ambert-Balay K, Botteldoorn N, et al.; NoroNet. Molecular surveillance of norovirus, 2005-16: an epidemiological analysis of data collected from the NoroNet network. Lancet Infect Dis. 2018;18:545–53. 10.1016/S1473-3099(18)30059-8 29396001

[6] Thanusuwannasak T, Puenpa J, Chuchaona W, Vongpunsawad S, Poovorawan Y. Emergence of multiple norovirus strains in Thailand, 2015-2017. Infect Genet Evol. 2018;61:108–12. 10.1016/j.meegid.2018.03.021 29597056

[7] Debbink K, Costantini V, Swanstrom J, Agnihothram S, Vinjé J, Baric R, et al. Human norovirus detection and production, quantification, and storage of virus-like particles. Curr Protoc Microbiol. 2013;31:15K.1.1–15K.1.45. 10.1002/9780471729259.mc15k01s31 PMC3920292 24510290

[8] Yang Z, Vinjé J, Kulka M. Complete genome sequence of human norovirus GII.Pe-GII.4 Sydney from the United States. Genome Announc. 2017;5:e00159–17. 10.1128/genomeA.00159-17 28408676 PMC5391414

[9] Botha JC, Taylor MB, Mans J. Comparative analysis of South African norovirus GII.4 strains identifies minor recombinant variants. Infect Genet Evol. 2017;47:26–34. 10.1016/j.meegid.2016.11.004 27833005

