# Supplementary material for: Recombinant GII.Pe-GII.4 Norovirus, Thailand, 2017–2018
Source: Emerg Infect Dis. 2019 Aug;25(8):1612–4. doi: 10.3201/eid2508.190365 (PMC6649319; doi:10.3201/eid2508.190365)
Supplement: Appendix — Phylogenetic tree of the norovirus GI partial nucleotide sequence of major capsid protein VP1, and cycle threshold values among patients in study of recombinant GII.Pe-GII.4 norovirus, Thailand, 2017–2018. [file 19-0365-Techapp-s1.pdf]

Article DOI: https://doi.org/10.3201/eid2508.190365

Recombinant GII.Pe-GII.4 Norovirus, Thailand, 2017–2018

Appendix

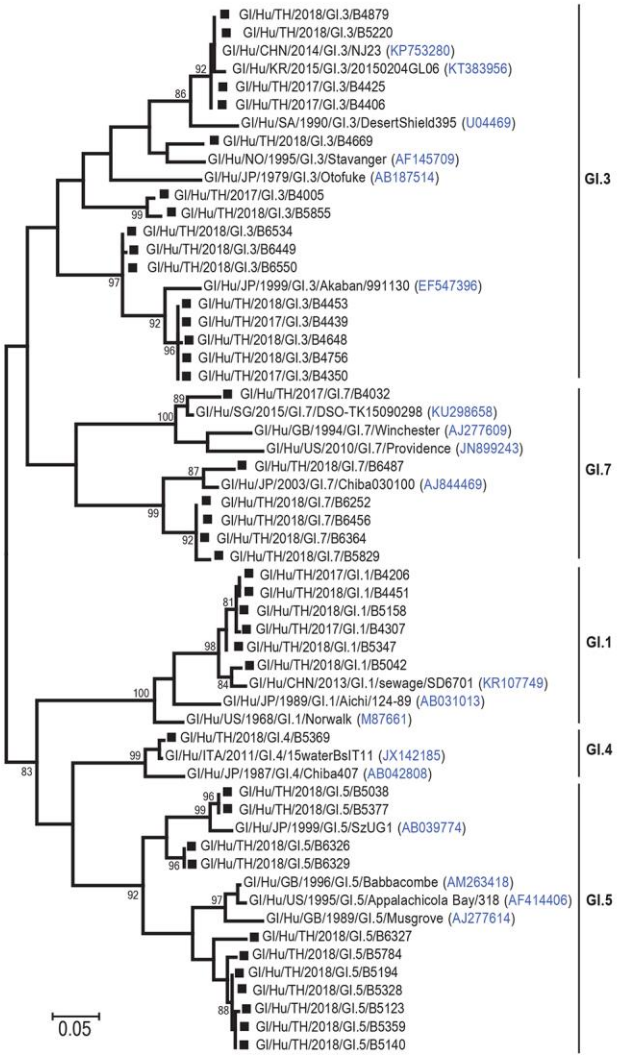

Appendix Figure 1. Phylogenetic tree of the norovirus GI partial nucleotide sequence of VP1. Trees were generated by using the maximum-likelihood method with 1,000 bootstrap replicates implemented in MEGA7 (https://www.megasoftware.net). Scale bar indicates nucleotide substitutions per site. Bootstrap values >80 are indicated at the nodes. Strains of sufficient nucleotide sequence length were included in the tree (denoted individually with squares). Reference strains are shown with accession numbers (parentheses).

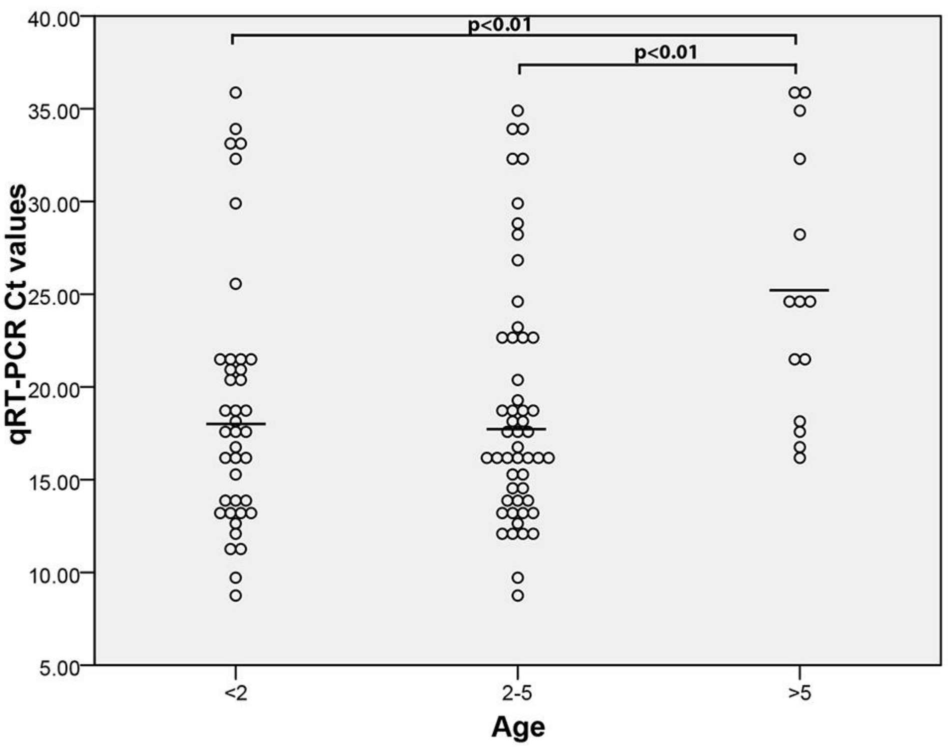

Appendix Figure 2. Box plot of the cycle threshold (C_t) values for GII.Pe-GII.4 Sydney among patients stratified by age groups. Bar in each column indicates the group mean value.
